# Physics-aware feedforward dwell time adjustment for mitigating distortion in additively manufactured cantilevers

**DOI:** 10.1007/s40964-025-01270-7

**Published:** 2025-07-30

**Authors:** Barış Kavas, Lars Witte, Efe C. Balta, Michael R. Tucker, Mohamadreza Afrasiabi, Markus Bambach

**Affiliations:** 1 ETH Zurich, Zurich, Switzerland; 2 Inspire, Zurich, Switzerland

**Keywords:** Laser powder bed fusion, Dwell time, Thermal simulation, Cantilever, Distortion

## Abstract

Heterogeneous temperature distributions in additively manufactured metallic parts, particularly in laser powder bed fusion (PBF-LB/M), pose a major challenge to achieving high-quality components due to thermal distortions, microstructural inconsistencies, and shifts in the process window. This study introduces a physics-aware feedforward approach for regulating dwell time that effectively mitigates distortion in 3D-printed cantilevers by reducing thermal variations along the build direction. A fast, 1D finite volume method thermal simulation is employed to estimate the temperature profile throughout the build. The interlayer dwell time is dynamically adjusted based on a predefined thermal difference threshold between layers to minimize residual stresses and part deformation. Experimental validation on a cantilever beam geometry confirms that the adaptive dwell time strategy significantly reduces distortion compared to a constant dwell time approach. The proposed method enhances thermal stability while maintaining processing times, offering an efficient solution for distortion control in PBF-LB/M. These findings contribute to advancing process optimization strategies by integrating physics-based thermal modeling with feedforward control.

## Introduction

Additive manufacturing (AM), particularly for metals, has revolutionized the production of highly complex parts that were previously difficult or impossible to fabricate with traditional manufacturing methods. By building up layers of material, AM enables the creation of complex geometries, internal features, lattice structures, and lightweight components with optimized topology and minimal waste [1]. Laser-based powder Bed Fusion (PBF-LB/M) is the most prevalent AM technology for metals, where parts are constructed by iteratively depositing layers of metal powder and selectively fusing them with a laser [2]. While it has been successfully adopted in many industries, significant processing challenges remain [3]. Particularly troublesome is the geometric distortion that can arise due to thermal gradients within the part.

### Overheating in PBF-LB/M

Process stability relies on maintaining a balance between the input, stored, and dissipated thermal energy. The energy input during laser exposure is determined by the cross-sectional area and process parameters, ensuring a stable melt pool. After exposure, residual heat dissipates through conduction, convection, and radiation—primarily through conduction into the underlying consolidated material and eventually to the build plate, as the surrounding powder acts as an insulator. When processing larger cross-sections, heat input per layer increases, while cooling remains limited, leading to *overheating* [4, 5]. Overheating negatively impacts print quality [6] by altering the substrate temperature, which affects melt pool size and solidification dynamics [7]. Shifting thermal boundaries lead to microstructural variations [8], and despite low oxygen levels, oxidation can still occur, resulting in discoloration and brittle oxide layers that hinder bonding [9]. Additionally, restricted heat dissipation causes temperature variations between layers, inducing thermal stresses and potential distortions [10, 11], which pose significant challenges to fully exploiting PBF-LB/M’s design freedom [12]. Strategies for mitigating thermal heterogeneity in PBF-LB/M generally fall into three categories: (1) optimizing part and support geometry; (2) tuning laser exposure parameters; and (3) adjusting dwell time. The first category proposes adjusting layer-wise cross-sections through design optimization [13], build orientation and layout modifications [14–16], and incorporating support structures [17, 18], which can help minimize thermal gradients and reduce overheating. However, these approaches often compromise the design flexibility and performance advantages of PBF-LB/M.

In the second category, heat input distribution is controlled by tuning process (or exposure) parameters such as scan path, sequence, velocity, and power to regulate melt pool stability and overall thermal management [19]. Tuning of the process parameters during the process based on the in-situ sensor data is called closed-loop control and has recently gained significant traction in the literature [20, 21]. Wang et al. [22] implemented a closed-loop PID controller to adjust laser power in situ, effectively reducing overheating-induced swelling. A similar type of controller is explored further and auto-tuned by Kavas et al. [23]. Their results indicate successful signal tracking at the expense of driving the laser power out of the nominal window, which results in porosity. Liao-Mcpherson et al. [24] developed a layer-to-layer closed-loop controller to stabilize the meltpool against varying geometrical conditions. Kavas et al. [25] developed a closed-loop control strategy to stabilize surface temperature, preventing overheating without additional cooling, but at the cost of reducing energy input below the fully dense process window.

Unlike feedback control, which dynamically adjusts parameters based on real-time measurements, feedforward control predefines process parameters using predictive models. Reiff et al. [26] employed an autonomous learning approach based on a thermal model to preemptively adjust laser power, reducing within-layer temperature variations by up to 48%. Boissier et al. [27] optimized scan paths using thermo-mechanical criteria, outperforming traditional linear scanning patterns. Liu et al. [28], Kim and Hart [29], and Qin et al. [3] developed alternative scan strategies that promoted more uniform temperature distributions and minimized distortion. Similarly, He et al. [30] optimized scan sequences to homogenize the thermal field and reduce deformation.

The studies mentioned above primarily focus on optimizing the heating cycle to minimize temperature variations and mitigate overheating. However, when part geometry and build layout are fixed, adjusting the heat input rate through adaptive dwell time strategies remains a more viable alternative. While tuning laser exposure parameters can improve temperature uniformity, the extent of improvement within a single layer cycle is inherently constrained by the fixed duration of exposures and recoating intervals [25, 31]. As a result, static recoating cycles hinder the ability to achieve temporally or spatially stabilized temperature profiles. Recent studies by Williams et al. [32] and Mohr et al. [33] highlight the influence of interlayer dwell time on 3D-printed part properties. Their findings show that shorter dwell times lead to elevated surface temperatures, promoting grain growth. Another work by Riensche et al. [34] demonstrated a feedforward approach that adjusts laser power and dwell time per layer based on the interlayer temperature change rate. Using part-scale thermal simulations, they achieved improved thermal stability and reduced overheating, highlighting dwell time as a key control parameter in PBF-LB/M. In a more recent study [35], Riensche et al. further refined the proposed control objective to stabilize the temperature history by adjusting multiple different process parameters. They showed a strong geometrical feature dependence of the IDT adjustment strategy on the microstructure.

Although these studies have demonstrated the potential of adjusting exposure and dwell time strategies to enhance thermal stability, they often lack a well-defined, transferable thermal target. This remains a significant gap in utilizing the dwell time assignment efficiently for the thermal management of various geometrical features. Therefore, the current literature does not fully exploit the potential of dwell time as an actuator for temporal thermal regulation to improve specific quality criteria of the printed parts. A fast and efficient temperature estimation approach is imperative to be utilized to evaluate the thermal management of the build volume.

### Thermal modeling approaches

Thermal modeling in PBF-LB/M follows three main approaches: analytical, empirical, and numerical. Analytical models, such as Rosenthal’s welding model [36], offer fast computations but rely on simplifying assumptions about boundary conditions, geometries, and material properties. Some more recent models account for heat accumulation [37] or part geometry [38], enabling efficient model-based optimization; however, they suffer from inaccuracies due to other simplified assumptions. Empirical models approximate experimental results and provide fast evaluations. Surrogate models, trained on simulated rather than measured data, significantly reduce computation time, with approaches like artificial neural networks (ANN) achieving speedups of three orders of magnitude for thermal simulations of fused filament fabrication [39], but require extensive training data and lack transferability. Numerical simulations, while more computationally demanding at various levels of fidelity [24, 40, 41], can capture details and provide insights that cannot be achieved through analytical or empirical approaches. At the part scale, methods such as finite element (FEM) [42, 43], finite difference (FDM) [44], finite volume (FVM), and particle-based techniques [45, 46] are employed, often incorporating adaptive discretization and efficient time-stepping to balance accuracy and speed.

The development of fast, physics-based, part-scale thermal models for PBF-LB/M remains an active area of research, with most studies focusing on multi-level adaptive discretization and parallel computing. State-of-the-art methods, such as those proposed by Leonor and Wagner [47] and Proell et al. [48], can generate scan-resolved temperature fields of an entire part without heuristic modeling assumptions like heat source scaling and agglomeration strategies. Nevertheless, these approaches still require several hours of computation for a 1$\times$1$\times$1 cm cube, making them impractical for iterative optimization, where hundreds to thousands of simulations may be needed within a single 1-h build job.

An alternative approach is reduced-order modeling. For instance, Peng et al. [49] developed an efficient thermal circuit network (TCN) model for a compound rectangular bar, leveraging the predominant heat conduction in the vertical (build) direction. They achieved a two-order-of-magnitude speedup while sacrificing a relative error of just 15 % compared to a 3D FEM model using the same simplified assumptions about boundary conditions. Subsequently, Peng et al. demonstrated fast distortion predictions based on the thermal history in 3D-printed disks with diameters ranging from 10 to 70 mm. However, multiple layers are combined within one element, which requires adapting the interlayer dwell time. In addition, the model assumes no radiative or convective heat transfer to the build chamber and neglects thermal conduction into the powder. Another promising approach involves linear 1D finite difference modeling with coarse horizontal discretization and implicit time integration, enabling simulation runtimes of less than 1-s [44]. Nonlinear effects, such as radiative heat transfer and temperature-dependent material properties, are omitted. Multiple layers are aggregated into a single element.

#### Uncertainty in process parameters and compensation strategies

Thermal models tend to be sensitive to process parameters that are often complex to measure and highly specific to the machine, material, and temperature. Key factors include laser absorptivity [50], material emissivity [51], bulk conductivity [52], and powder conductivity [53]. Accurately measuring surface or part temperatures is particularly challenging due to steep spatial and temporal gradients. In-situ thermal imaging systems, such as thermal cameras and pyrometers, require careful calibration tailored to specific materials and processes. These uncertainties impact strategies for distortion compensation and process control. One approach involves geometry adjustments based on part-scale distortion simulations [54], while another focuses on optimizing process parameters to minimize overall distortion. Interlayer temperature control has been demonstrated using a fast finite difference model [44], where laser power is the primary control variable. Experimentally identified controller models have also been tested by varying laser power; however, relying solely on laser power has been shown to be insufficient for stabilizing interlayer temperature [25]. Therefore, the literature lacks a simple yet efficient method that addresses the parameter adjustment problem for improving various build quality metrics.

### Present work

From the reviewed literature, two critical research gaps emerge that motivate the present work. First, while recent studies demonstrate the impact of dwell time on microstructure and thermal stability, no transferable framework currently exists for assigning dwell times based on an efficient and real-time applicable criterion. Most thermal control approaches ignore dwell time as an actuation input and assume a fixed dwell time assignment throughout the build. This limits their ability to adapt to varying geometries or local heat accumulation effects.

Second, existing adaptive process parameter strategies—particularly those integrating feedforward control—often employ high-fidelity thermal simulations that are computationally expensive. While accurate, these models are impractical for real-time or iterative optimization across full build volumes. There remains a pressing need for employing simplified, yet reliable and flexible, thermal estimation models that balance speed and accuracy, enabling fast control decisions at the part scale.

Therefore, the present work seeks to address the following open questions:

- How can a geometry-aware, temperature-difference-based metric be used in a feedforward strategy to adaptively control dwell time and thereby reduce thermal distortion in PBF-LB/M?
- How can a computationally efficient yet physically representative thermal model be developed to enable adaptive thermal control in PBF-LB/M without resorting to high-fidelity simulations?

To address the identified research gaps and questions, this study proposes a novel feedforward control strategy for PBF-LB/M that adaptively adjusts dwell time based on interlayer temperature differences. A temperature-difference-based thresholding method is introduced to guide dwell time assignment, and its effectiveness in reducing thermal distortion is demonstrated on a cantilever beam structure. A one-dimensional finite volume thermal model is also developed to estimate the vertical temperature profile during the build.

The remainder of this manuscript is structured as follows. Section 2 details the experimental and computational frameworks. Section 3 present the simulation and experimental findings, followed by an in-depth discussion in Sect. 4. Conclusions and future directions are outlined in Sect. 5.

## Materials and methods

This section describes the proposed approach, the simulation methodology and its implementation, as well as the experimental setup and procedure used to evaluate the improvements. A schematic overview of the experimental and simulation framework is provided in Fig. 2.

### Proposed approach for adjusting dwell time

To mitigate thermal distortion caused by steep thermal gradients in the vertical build direction, the proposed approach introduces an artificial waiting period, referred to as the interlayer dwell time (IDT), at the end of each layer.

**Fig. 1:**
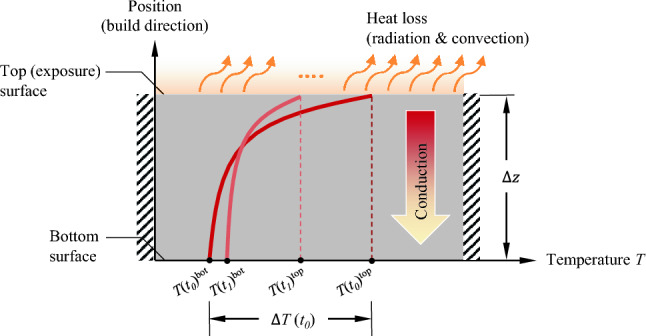
Schematic of heat transfer across a solid part cross-section: conduction from new layers, surface cooling, and temperature evolution over time. The lateral sides are assumed to be adiabatic. The temperature profile shortly after laser exposure is represented by the curve $t_0$ (dark red), while the $t_1$ curve (light red) depicts the temperature profile after some time. the $\Delta {z}$ describes the distance between the latest printed layer and the lowest layer of the cross-section

The local thermal conditions are modeled using a 1D element representation. The post-exposure cooling behavior is conceptually illustrated in Fig. 1, where $T(t_n)$ represents the temperature distribution at discrete time steps *n*, with $n \in \mathbb {Z} \cap [0, \infty )$ and *t* denoting the elapsed time after exposure.

As discussed in Sect. 1, excessive temperature gradients negatively impact part quality. Therefore, limiting the temperature difference between the top and bottom surfaces, $\Delta T^{tb} = T(t_n)^{\text {top}} - T(t_n)^{\text {bot}}$, was chosen as the control objective, achieved through adaptive IDT assignments. Determining the IDT based on the temperature difference for each layer requires access to both $T(t_n)^{\text {top}}$ and $T(t_n)^{\text {bot}}$.

It is important to note that the thermal stress is the result of the immediate post-solidification cooling temperatures, where the high-temperature gradients in both time and space are observed [55]. The proposed method leverages the spatially averaged temperature over a layer with the uniform and homogenous heat input assumption concerned with the temperature and cooling ranges much later than the solidification event. Therefore, residual stress reduction is not directly targetted by this technique, but indirectly through the reduction of temperature difference before the next exposure application. This also allows a more accessible type of data, e.g. less spatially and temporally demanding in terms of sensor hardware and thermal gradients to be used for model fitting.

While process monitoring systems can directly measure $T(t_n)^{\text {top}}$, obtaining $T(t_n)^{\text {bot}}$ typically requires estimation via thermal modeling. In this work, the adaptive IDT duration for each layer is determined before the fabrication in an offline computation step by a threshold-based approach: an FVM-based thermal simulation computes $T(t_n)^{\text {top}}$ and $T(t_n)^{\text {bot}}$ over time until the predefined $\Delta T_{tb}$ threshold is met.

**Fig. 2:**
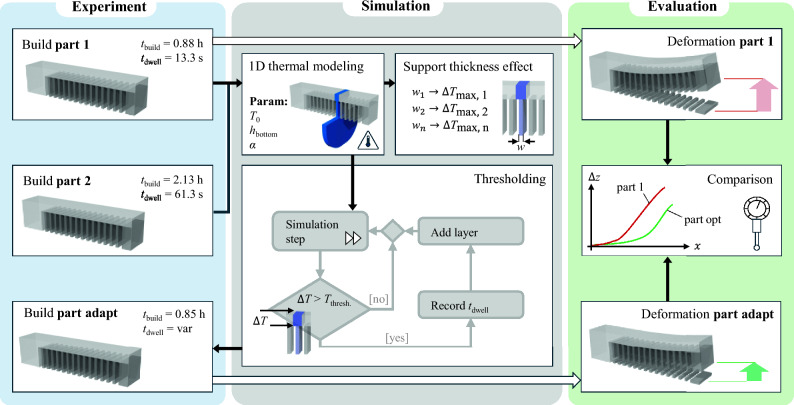
Schematic overview of the experimental, simulation, and evaluation workflows. The experimental process (left) involves different builds. Reported dwell and build times ($t_{\text {dwell}}$ and $t_{\text {build}}$) correspond to a part with a *Z*-height of 7.2 mm. The simulation block (middle) incorporates 1D thermal simulation and is based on the modeling, determining the adaptive dwell time by thresholding, and investigation of the support thickness on temperature differences in the *z*-direction. The evaluation procedure (right) compares deformation results between the build of **part 1** with constant waiting time and build **part adapt** with adaptive waiting times

### Part-scale thermal simulation

In the PBF-LB/M process, local heat transfer in the vertical plane of the part can be approximated using a unidirectional heat flux assumption. This allows for an explicit FVM simulation to predict the spatial temperature distribution by modeling heat transfer through the layers. The primary motivation for simplifying the model to 1D heat transfer between elements is to enable fast computation of the local temperature profile along the *z*-direction (vertical), which captures the dominant physics for the application at hand. By employing relatively large elements and omitting detailed laser scan paths, the number of elements remains low, allowing for larger time steps and reduced computational cost.

**Fig. 3:**
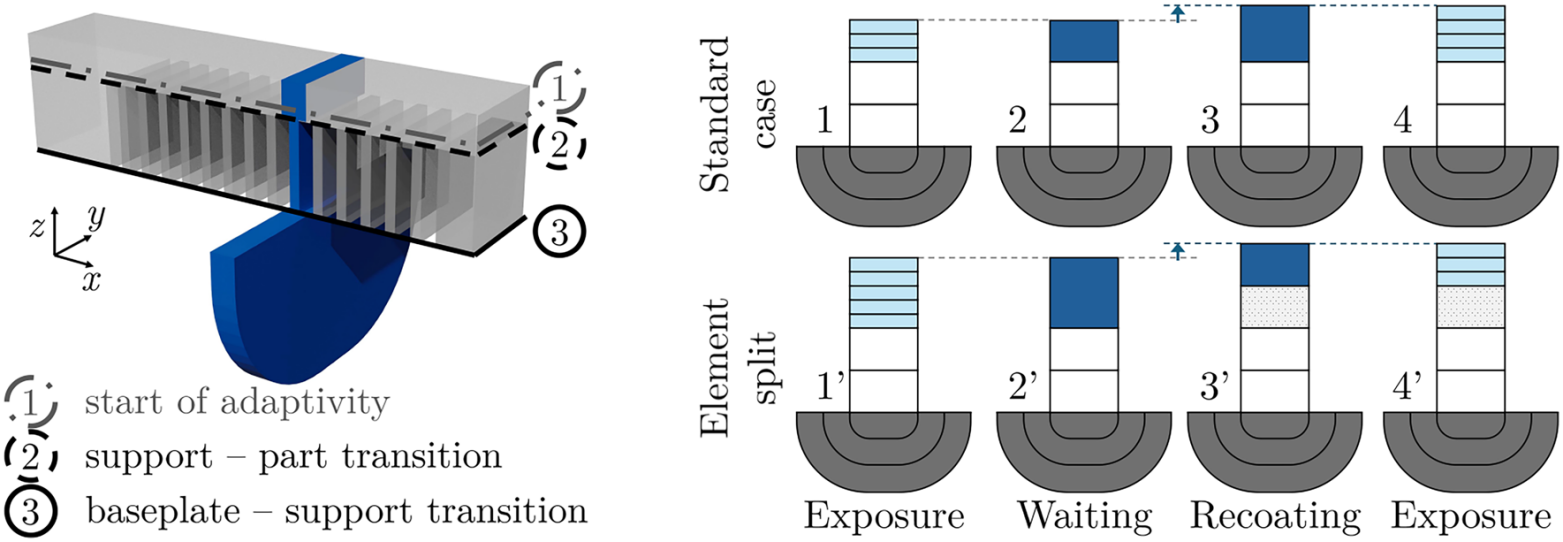
3D rendering of the cantilever beam (translucent grey), with the modeled 1D element slice (blue) positioned near the center (left). Schematic representation of the spatial adaptivity technique (right)

A cantilever beam was chosen as a representative example (Fig. 3 (left)). Due to the periodic nature of the support structures, adiabatic conditions were assumed in the longitudinal (*x*) direction near the middle of the part. The modeled section was centered on one support and extended half the distance to the neighboring supports on either side in the *x*-direction. The top element represents the beam, spanning the segment width in the *x*-direction and the beam depth in the *y*-direction. Heat spreading into the base plate was modeled primarily as radial due to the assumed adiabatic conditions in the *x*-direction. The bottom plate was discretized such that heat conduction remained approximately normal to the element boundaries.

#### Discretization method

The simulation follows a numerical FVM approach, solving the standard heat equation:

1 $$\begin{aligned} \rho c_p \frac{\partial T}{\partial t} = \nabla (k \nabla T) + \dot{p}_V, \end{aligned}$$

where $\rho$ is the material density, $c_p$ the specific heat capacity, and *k* the thermal conductivity. Here, *T* represents temperature, *t* is time, and $\dot{p}_V$ denotes the heat source as power per unit volume.

The temperature evolution of each element is computed as:

2 $$\begin{aligned} V^e \rho c_p \frac{\textrm{d}T^e}{\textrm{d}t} = \sum _{i} P_i^e, \end{aligned}$$

where $V^e$ is the element volume, and the sum $\sum _{i} P_i^e$ accounts for heat fluxes due to conduction between neighboring elements, boundary interactions, and laser heating. Although this approach allows for temperature-dependent material properties, a constant property assumption was used due to the relatively low temperatures in the present test cases.

#### Boundary conditions

The heat flux at the top surface is given by:

3 $$\begin{aligned} P^{\text {top, BC}}= & D P_{\text {laser}}(t) - A^{\text {top}} \left[ h_{\text {conv}}\left( T^{\text {top}} - T_0\right) \right. \nonumber \\ & + \left. \varepsilon _r \sigma _{\text {SB}}\left( {T^{\text {top}}}^4 - {T_0}^4\right) \right] , \end{aligned}$$

where $P^{\text {top, BC}}$ is the total heat flux into the top element. During laser exposure, the element absorbs laser power $P_{\text {laser}}(t) > 0$, scaled by the total absorptivity *D*, which accounts for both the material’s standard absorptivity and additional heat losses, such as radiation from the high-temperature melt pool and evaporation effects. Convective heat loss is modeled using the heat transfer coefficient $h_{\text {conv}}$, while radiation effects are captured through the emissivity $\varepsilon _r$ and the Stefan-Boltzmann constant $\sigma _{\text {SB}}$.

At the bottom surface, thermal resistance was introduced to model heat dissipation into the base plate, which was assumed to have a constant initial temperature $T_0$. The small, semi-enclosed powder volume between the supports justified the assumption of adiabatic conditions on the powder-contacting surfaces. A heat transfer coefficient $h_{{\textrm{conv}\_{\textrm{side}}}}$ was applied to the vertical powder-contacting surfaces in the *y*-direction, while the powder itself was modeled with a constant initial temperature.

#### Time integration and adaptivity

Time integration was performed using an explicit fourth-order Runge–Kutta (RK4) method. Excluding logging, the simulation time scaled approximately linearly with the number of time steps required. According to the Courant-Friedrichs–Lewy (CFL) criterion for the Euler method and parabolic PDEs [56]:

4 $$\begin{aligned} \Delta t < \frac{{\Delta x}^2}{2\alpha } \end{aligned}$$

a larger element size suggests a quadratic increase in the maximum stable time step. While this criterion does not directly apply to RK4, it provides a useful estimate, indicating that larger elements can significantly increase the stable time step length.

Since strong spatial and temporal gradients primarily occur during and immediately after laser exposure, and are mostly confined to the upper layers, a coupled adaptivity scheme for element size and time step length was implemented, as schematically illustrated in Fig. 3 (right). Two different cases occurring depending on the z-height are illustrated with two examples: the standard case and the element split case. States 1 and 1$'$ depict the system schematically during laser exposure and shortly after ($t_{\text {res}}$), where fine, layer-sized elements (light blue) of height *l* are used at the top, while the time step length is reduced by a factor of $10^2$. Coarse elements of height $l_a$ are positioned below to model support, part, or both (white). Bottom plate elements are schematically shown in grey. After the high-resolution time $t_{\text {res}}$, all fine top elements merge into a single larger element (dark blue), as shown in states 2 and 2$'$, allowing the time step length to be increased by the same factor of $10^2$. Before the subsequent exposure, the element size increases by the layer height *l* as illustrated in step 3 (standard case) due to recoating. The only exception occurs when the new thickness of the top element (dark blue) would be twice the thickness of the coarse elements ($l_a$); in that case, the top element is split into two, illustrated in state 3$'$ (element split case), ensuring top element sizes are always smaller than $2l_a$. After the split, all support/part elements have a height of $l_a$. In states 4 and 4$'$, the top elements are refined again, marking the start of the next exposure cycle.

#### Implementation

The model was implemented in Python and NumPy. A simulation consisting of 333 layers, each 30 $\upmu$m thick, with a layer-to-layer time (including scanning, recoating, and interlayer dwell time) of 23 s per layer (based on the parameters in Table 1) runs in under one minute on an Intel i9-13900K processor. This corresponds to a speedup of approximately 125 times compared to real-time.

The average waiting time per layer was chosen to match the physical experiment (see below), incorporating recoating time along with an additional 10 s of IDT.

**Table 1:** Layer and time step parameters

Parameter	Symbol	Value	Unit
Layer height	*l*	0.03	mm
Coarse adaptive element height	$l_a$	0.3	mm
Time step small	$t_s$	0.1	ms
Time step large	$t_l$	10	ms
Total high resolution time	$t_{\text {res}}$	500	ms
Exposure time support	$t_{\text {sup}}$	80	ms
Exposure time part	$t_{\text {part}}$	200	ms

During the exposure, the energy input to the top element of the simulated part section by the laser is modeled as shown in Eq. (3) without including a moving laser path through the duration of the laser exposure for the layer. The exposure times were calculated based on the time required for the laser to expose the support/part section, considering the experimental hatching distance ($0.1 \, \text {mm}$) and the laser velocity ($800 \, \text {mm/s}$).

The material properties of 316 L stainless steel were assumed to be constant due to the relatively low simulated temperatures during solid-state conduction. Thermal conductivity ($15 \, \text {W/mK}$), specific heat capacity ($500 \, \text {J/kgK}$), and density ($8000 \, \text {kg/m}^3$) were taken at $20^\circ C$ from [57]. An emissivity value of 0.4 was chosen based on [58].

The heat transfer coefficient into the powder, $h_{{{\textrm{conv}}\_{\textrm{side}}}}$, was estimated at $8 \, \text {W/m}^2\text {K}$ based on an approximation where the effective thermal conductivity of the powder is assumed to be 1% of the solid conductivity, with a width of 12 mm, following [59]. The convection coefficient for the top surface, $h_{\text {conv}} = 16.814 \, \text {W/m}^2\text {K}$, was approximated using the method from [60], considering forced convection over a flat plate with an unheated starting length of 0.45 m, an airspeed of 1 m/s, and a part length of 0.01 m.

### Experimental setup

A fixed cantilever beam geometry was selected for the experimental validation of the proposed method, with two beams printed using the same process parameters per build. A laterally positioned thermal camera captured the surface temperature of the parts at various time points during fabrication.

Three experiments were initially conducted, each with different constant IDT of 0, 10, and 60 s per layer, to assess the influence of IDT on part distortion. Temperature measurements from the 10-s and 60-s IDT builds were used to optimize the simulation parameters, as detailed in Sect. 3. To ensure a fair comparison between constant and adaptive IDT, a heuristic optimization of the $\Delta T_{sb}$ threshold was performed to generate an IDT assignment trajectory approximating the total IDT duration of the 10-s constant IDT part. Finally, a build incorporating dynamic IDT assignment following the optimized trajectory was carried out using the same cantilever beam design. Additionally, the simulation framework was applied to cantilever beam designs with varying line support thicknesses to investigate the effect of support structures and further demonstrate the method’s applicability.

#### Specimen design

**Fig. 4:**
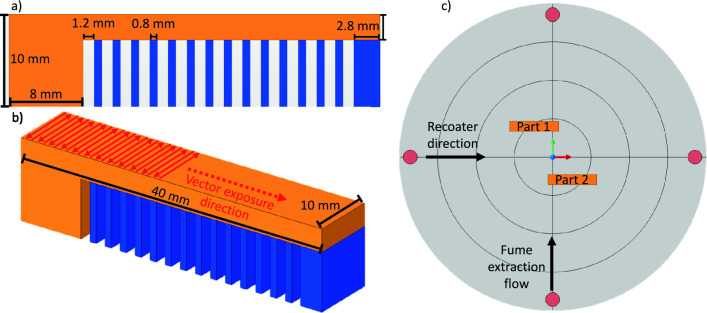
Geometrical parameters and build platform placement of the simulated and 3D-printed cantilever beams

The cantilever beam geometry is illustrated in Fig. 4. The orange region represents the cantilever beam, whereas the blue region corresponds to the support structures. The beam measures 32 mm in length, 2.8 mm in thickness, and 10 mm in depth, resting on 7.2 mm tall supports. A solid block at one end ensures a rigid connection to the build plate. The same process parameters were applied across all regions within a single build. A serpentine scan strategy was used for both the beam and supports, with hatch progression starting from the fixed block and extending toward the supports in all layers to maximize residual stress accumulation (Fig. 4b). No interlayer hatch rotation was applied.

With a layer thickness of 30 $\upmu$m, the upper beam begins at layer 240 and requires 93 layers for completion. The parts were positioned on the build plate as shown in Fig. 4c, with *Y*-axis offsets to avoid (or minimize) interactions with the recoater and spatter.

#### PBF-LB/M processing

The experiments were conducted on an Aconity3D Midi+ (Aconity3D GmbH) PBF-LB/M system [61]. The machine was equipped with a continuous-wave Gaussian-mode fiber laser (nLIGHT Alta) operating at a wavelength of 1080 nm. The laser was focused to a beam diameter of $d4\sigma = {80}\,\upmu \hbox {m}$, with a layer thickness of $l = {30}\,\upmu \hbox {m}$. To achieve a fully dense microstructure, nominal process parameters of 140 W laser power and an 800 mm/s scan speed were applied. The powder used in this study was gas-atomized stainless steel 316 L (1.4404) with a particle size distribution of $15-{45}\,\upmu \hbox {m}$ (CT POWDERRANGE 316LF, Carpenter Additive). The nominal recoating time was 13 s.

#### Measurement

**Fig. 5:**
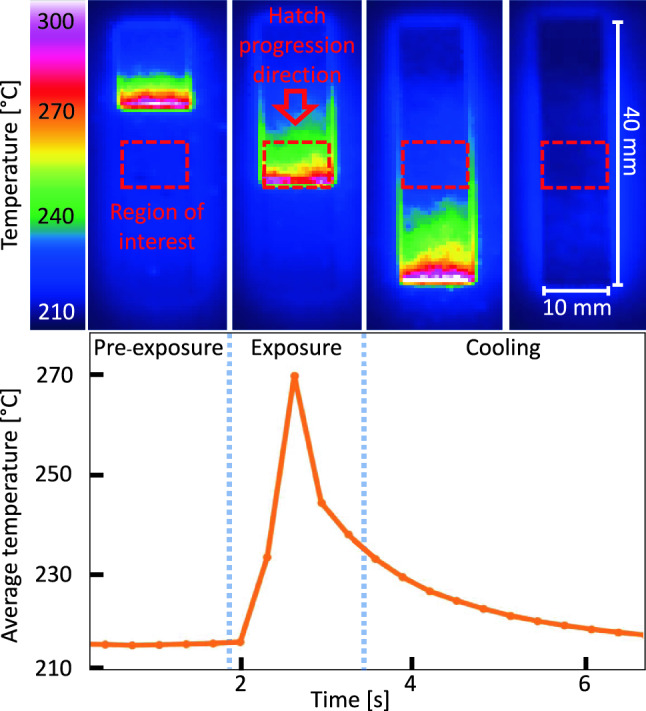
Top: thermographic snapshots at four representative instants during the processing of a single powder layer, with laser exposure visible in the first three frames. Bottom: corresponding time-series of spatially averaged substrate temperature, showing the peak thermal response during laser irradiation and the subsequent powder-induced cooling phase used for model calibration

The PBF-LB/M system has an Infratec HD800 thermal camera placed laterally above the the build platform. During the experiments, each layer exposure and the following cooling time is captured through a video recording. Due to the spatial resolution limitations, the temperature of the meltpool and its vicinity are not possible to be accurately measured, nor of direct interest to this study. Therefore, the region of interest is selected from the central location of the beam, and the temperature reading is registered as a spatially averaged value for the time of measurement as shown in Fig. 5.

The upper section of the figure shows four instances during the processing of a layer, where the laser exposure can be seen in the first three. The corresponding averaged temperature measurements are also shown as a time-series plot. The effect of powder and the resulting cooling after the exposure is shown, as the cooling information is used for the model calibration.

**Fig. 6:**
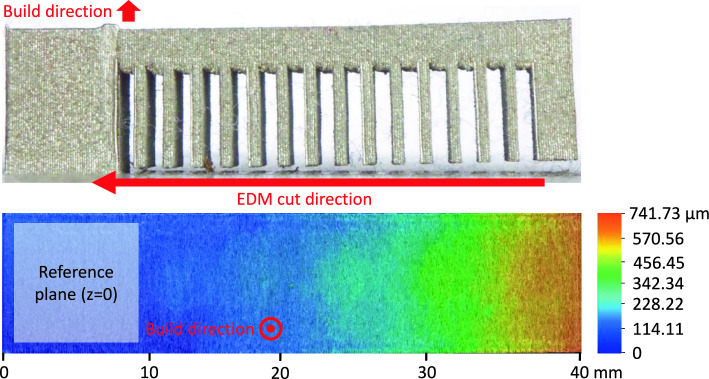
Partial cutting of the cantilever beam from the supports and an example of upper surface height profile measurement

After completion, the build platform was removed from the PBF-LB/M machine and cleaned of residual powder. The parts were then partially cut using electrical discharge machining (EDM) from the support side up to the fixed block section of the main part, as shown in Fig.6 (top). This approach is commonly employed to quantify residual stress within the upper beam by releasing it in the form of distortion [30]. In the partially cut state, the beam’s upper surface height was measured relative to a reference plane, defined at the top of the fixed block, along evenly spaced lines perpendicular to the beam’s longitudinal axis (Fig. 6, bottom).

Measurements were performed using a Keyence VHX-7000 digital microscope in coaxial and ring lighting modes with 100x magnification. The 3D scan data was corrected for any tilt relative to the reference plane (Fig. 6, bottom-left), assuming minimal distortion in the fully fixed section. The height profile was obtained by averaging the measured values along the cross-sectional axis, and the resulting profile was plotted against the longitudinal position for comparison and evaluation.

## Results

This section presents the calibration of model parameters, the impact of L2L time adaptivity by thresholding on time and temperature results, the influence of varying support thicknesses, and a comparison of experimental distortions between adaptive and constant IDT assignments.

### Model parameter calibration

Experiments were conducted using two different layer-to-layer times: 33.6 s and 81.6 s, during which surface temperatures were measured. The absorption coefficient, preheating temperature, and base plate thermal resistance were calibrated using an inverse approach. A tree-structured Parzen Estimator algorithm optimized these parameters over 200 iterations, minimizing a mean absolute percentage error (MAPE) loss function (Eq. (5)). To mitigate inaccuracies near the support-part transition, the loss calculation was performed starting 600 $\upmu$m above this interface in the *z*-direction.

The bottom BC predominantly depends on the bottom plate geometry. Literature reports a 316 L absorption coefficient of approximately 0.6 under a 150 W laser power, 1500 mm/s scanning velocity, a 100 $\upmu$m powder layer, and a 1070 nm laser with a $60\pm 5$ $\upmu$m 1/$e^2$ Gaussian beam diameter [50]. The inverse parameter determination resulted in the values summarized in Table 2.

**Table 2:** Inversly determined simulation parameters

$T_{\text {0}}$	BC bottom	Absorption coefficient
$20.13 \ ^{\circ }\text {C}$	98.35 $\hbox {W/m}^{2}\,\hbox {K}$	0.4099

**Fig. 7:**
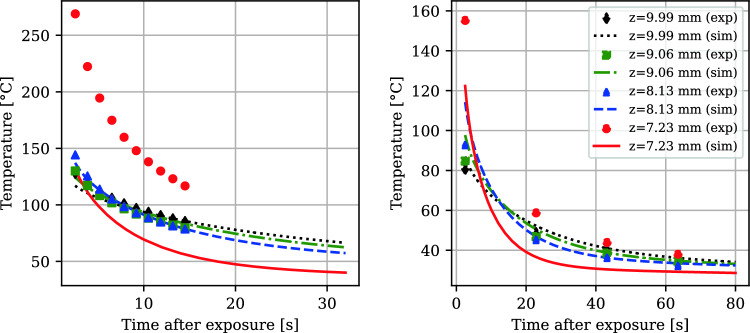
Comparison of simulation results with the experimental data at different layer-to-layer times: 33.6 s (left) and 81.6 s (right). The support top (beam bottom) is at *z* = 7.2 mm. The red data points and simulation plot indicate lower accuracy obtained at the initial layers

Comparison of experimental surface temperature cooldowns with simulation results at different heights showed good agreement, except for the initial layers of the cantilever. Figure 7 shows this trend.

MAPE values were computed for each layer using:

5 $$\begin{aligned} \text {MAPE} = \frac{1}{N}\sum _{i=1}^{N}\left| \frac{T_{\text {exp}}^i - T_{\text {sim}}^i}{T_{\text {exp}}^i}\right| \end{aligned}$$

**Fig. 8:**
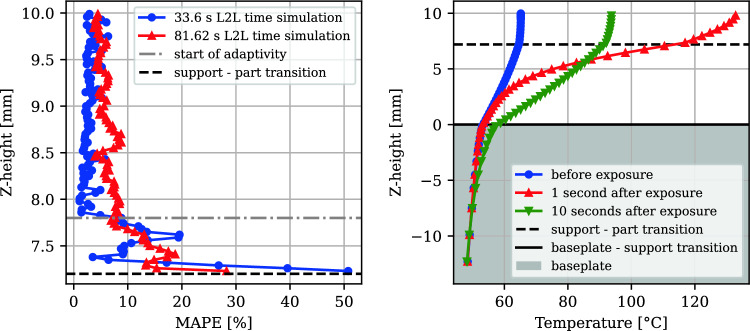
MAPE error between simulation and experimental surface temperatures (left). Temperature profile in the *z*-direction over time after exposure during the 33.6 s run (right). Each point corresponds to the temperature of one element measured before exposure, 1 s after exposure, or 10 s after exposure. The baseplate elements are at $z<0$

Above a *Z*-height of 7.8 mm, the MAPE remained below 10%. Thresholding for adaptive waiting time determination, based on the *z*-temperature profile, was initiated at this height by calculating the temperature difference between the top and the support-part transition—see the right plot in Fig. 8.

### Sensitivity of MAPE to variations in calibrated parameters

**Fig. 9:**
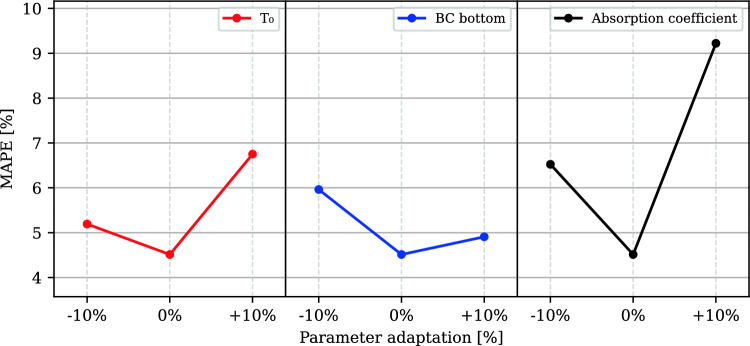
Sensitivity of the MAPE error to variations in the calibrated parameters. While one parameter was varied, the others were held constant at their calibrated values

Varying the calibrated parameters by ± 10% resulted in higher average MAPE for all calibrated parameters (Fig. 9). The largest increase in MAPE was observed with adaptation of the absorption coefficient, whereas the smallest increase was noted with adaptation of the bottom heat resistance, given the same relative parameter variation.

### Adaptivity by thresholding for dwell time adjustment

**Fig. 10:**
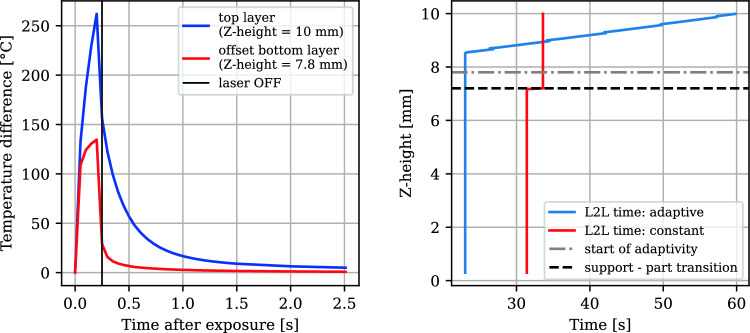
Temperature difference between top and bottom layers over time (left). L2L time distribution across support and part heights (right)

Adaptivity of L2L times by thresholding was applied above layer height 7.8 mm, as indicated in Fig. 8 (left) by the “start of adaptivity” dashed line. The temperature difference between the top and bottom layers was used as a criterion (the left plot in Fig. 10). If this difference fell below $0.09\ ^{\circ }\text {C}$, the next exposure was scheduled, enabling a feedforward determination of the dwell time profile as shown in Fig. 10 (right) before fabrication. The adaptive L2L times start with the 24 s minimum possible L2L time for the experimental setup during part exposure. Due to the shorter exposure time at the support Z-heights compared to the part heights, the constant L2L time build has slightly reduced support L2L times (part: 33.6 s, support: 31.4 s). This also applies to the second build (part: 81.62 s, support 79.42 s).

**Fig. 11:**
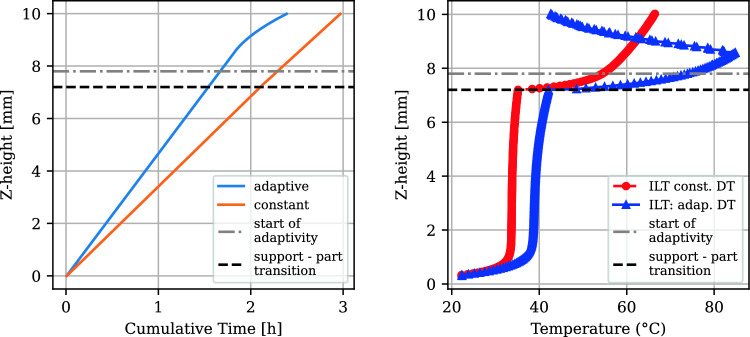
Cumulative processing time comparison of constant with the thresholded process (left). Simulated ILT comparison (right)

Adaptive waiting times resulted in an average of 33.28 s per layer in the part, slightly reducing the total processing time compared to the constant 33.6 s scenario (Fig. 11). The resulting distortion was reduced by 22%, demonstrating the effectiveness of adaptive L2L times in minimizing deformation. In the adaptive build, the overall cumulative time was 19.6% shorter than the constant L2L time build (Fig. 11 left). The cumulative processing time of just the part (time spent over the support-part transition) in the adaptive L2L time build was 2.6% shorter than in the constant dwell time build. The interlayer temperature (ILT) is influenced by changed dwell time distribution as shown in Fig. 11 (right). This surface temperature, measured directly before the next exposure, increases during the constant dwell time build across the part. In the thresholded dwell time build, it first increases and then decreases.

### Support thickness design

**Fig. 12:**
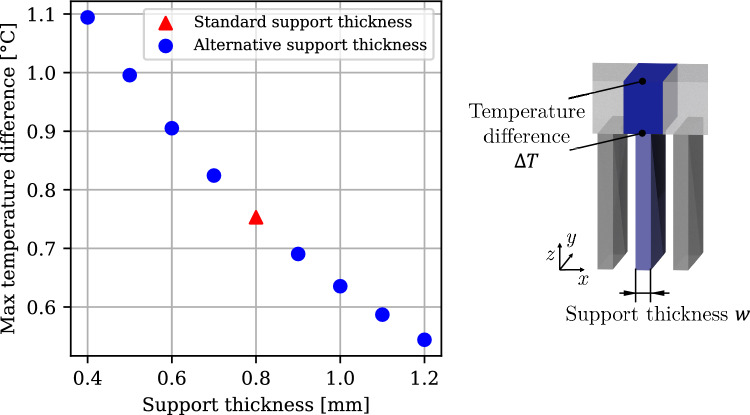
Influence of support thickness on the maximum simulated top-to-bottom temperature difference immediately before the next exposure

Based on the hypothesis, support thickness can be optimized to minimize waiting time and mitigate distortion effects. For a layer-to-layer time of 23 s, various support thicknesses were simulated, and the corresponding maximum thermal differences are presented in Sect. 3.4 (Fig. 12).

### Experimental observations

**Fig. 13:**
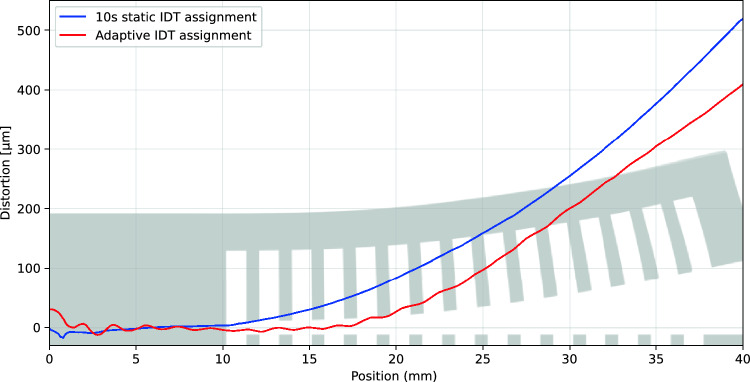
Optically measured deformation in controlled and uncontrolled cantilever beams

Figure 13 presents the deformation profiles after support removal for both the adaptive and constant L2L time builds. The shaded cross-sectional figure in the background provides an illustrative reference for the longitudinal position on the *X*-axis. To enhance clarity, both curves have been smoothed using a rolling average with a window size of 4000.

The slight waviness observed in the adaptively assigned part’s profile (red) has also been qualitatively identified at higher magnification. This waviness likely stems from a minor recoating error, which appears to have no significant correlation with the thermal distribution of the part. The deformation profiles remain relatively flat in the fully connected region (up to 10 mm), after which a pronounced distortion occurs in the beam section. This distortion results from stress relief due to the disconnected supports. While both profiles exhibit a similar overall pattern, the tip displacement is measured at 409 $\upmu$m for the adaptively IDT-assigned part and 519 $\upmu$m for the constant IDT-assigned part, demonstrating a reduction in distortion with adaptive support assignment.

## Discussion

### General approach

The experimental results demonstrate that the proposed thermal criterion effectively reduces residual stress accumulation in a cantilever beam geometry (Fig. 13), leading to a 22% reduction in tip distortion upon support removal. The proposed method is based on well-established studies in the literature that link temperature differences within the printed part to the thermal stress accumulation during the printing process. This association allows the proposed method to employ a simple-to-measure and model temperature criterion for reducing the resulting distortion, as validated with a canonical cantilever beam geometry that is commonly used for residual stress quantification. Literature provides a well-established understanding of thermal stress buildup during PBF-LB/M, however, a direct link between the proposed temperature criterion and deformation mechanisms would require a more rigorous thermomechanical model, which is left as future work.

Compared to previous studies that employ complex thermo-mechanical models to reconstruct the PBF-LB process step by step, our results suggest that thermal distortions can be reduced without explicitly calculating the thermo-mechanical response of the entire geometry. The model-based adaptive L2L time approach offers an efficient and effective cooling actuation strategy, requiring only process monitoring for tuning the model’s physical parameters.

In this study, the benchmark and controlled parts share identical geometries and the thermal threshold is selected to maintain equal total build durations. The improved distortion profile in the controlled part demonstrates that inherent dwell times, such as exposure of other regions within a layer, can be strategically designed based on thermal criteria to enhance build quality without extending the total build time.

The scan strategy employed in this study was intended to generate high residual stresses in cantilever beam geometries. Specifically, it features sequential exposure progressing linearly across each layer, employs a non-alternating hatch direction consistently throughout layers, and initiates hatching from unsupported (foot) towards supported regions, enhancing within-layer thermal gradients. Consequently, more common scan strategies would likely result in lower residual stress buildup [30]. The performance of the proposed method over the thermally suboptimal scan strategy conservatively suggests that the thresholding method could also perform well for more thermally convenient scan strategies like hatch alternation, striping, islands, and others. Together with the proposed method itself being independent of the scan strategy, the quantitative improvement observed in practice may vary accordingly.

The proposed method makes a notable contribution to dwell time adjustment strategies by offering a practical and computationally efficient alternative to traditional energy input optimization. While tuning laser parameters is a common approach to mitigate thermal buildup, its effectiveness is fundamentally limited by the thermal balance constraints of the process. Artificial dwell times, though often avoided due to their impact on build duration [62], remain the only direct lever for regulating energy transport across layers.

Riensche et al. [34, 62] employs a graph-based model for thermal control. In contrast, our approach utilizes an efficient 1D finite volume model without super-layer aggregation to adapt dwell time-based on interlayer temperature differences, enabling straightforward offline computation and easing real-time implementation. While their use of super-layer aggregation reduces computational load, it also limits temporal granularity, as opposed to the method proposed in this study, which trades generality for simplicity and achieves faster computation times per individual simulated layer.

Besides the employed simulation approach, the proposed specific thermal target based on the temperature difference between two surfaces is complementary to the stabilization objective that they assigned. Feed-forward dwell time adjustment utilizing a single dwell time change during the build process was demonstrated [34]. Constant layer-to-layer times, like in our reference approach, were tested for samples with variations in layer exposure times by adjusting the dwell time of each layer to compensate [62]. Our adaptive approach implements feed-forward dwell time control on a layer-by-layer basis and reduced distortion for the cantilever geometry compared to a reference approach with the same average but constant layer-to-layer time in the beam section.

While closed-loop control schemes—such as those by Kavas et al. [23, 25]—and other power-adjusting methods like Reiff et al. [26] offer fine-grained thermal regulation, they introduce significant implementation complexity. These methods rely on real-time sensor feedback, extensive calibration, and complex signal processing pipelines, and they may require specialized hardware such as field-programmable gate arrays. Additionally, they risk driving the laser power outside the nominal process window, potentially inducing defects such as porosity.

Reiff’s approach, for example, uses a thermal model to modulate laser power within layers, effectively compensating for intra-layer temperature variations. However, this comes at the cost of system complexity and tight integration with real-time data streams. In contrast, our feedforward dwell time strategy operates at the interlayer scale and relies exclusively on precomputed thermal thresholds. It eliminates the need for real-time sensing and complex hardware, resulting in a substantially simpler and more robust solution for industrial deployment—especially in environments where closed-loop infrastructure is impractical or unavailable.

Importantly, our method offers several practical advantages: when dwell times are assigned in advance, overall build time becomes predictable, and no thermal camera is required for deployment. In our case, the thermal camera was used solely for offline model calibration and validation. Once calibrated, the model can be used directly, provided that its assumptions hold and the geometry-specific parameters remain valid.

While feedforward control lacks adaptability to unexpected disturbances, unmodeled thermal phenomena, or poor model fits, it proves effective for managing overheating behavior that can be anticipated in advance. By merging temperature estimation through modeling with cooling control via dwell time adjustment, the proposed strategy combines computational efficiency with implementation simplicity. Compared to the baseline cantilever beam printed with uniform timing, our method demonstrates significant potential for scalable, robust control—offering a compelling path forward for industrial additive manufacturing applications.

Previous studies have demonstrated the influence of dwell time on microstructure. Williams et al. [32] and Mohr et al. [33] reported that shorter dwell times can elevate surface temperatures, leading to grain coarsening or porosity. Riensche et al. [62] showed that stabilizing the IDT significantly reduced grain size variation in non-overhanging geometries. These findings underscore the well-established role of IDT in microstructural control. The proposed method, which enables control over the 1D temperature distribution, offers a means to induce specific cooling patterns that support stable or tailored part-scale microstructures. Thus, adaptive dwell time strategies present a promising avenue for future research in microstructural optimization.

Although using physics-based models for pre-assigning the process parameters is not new to manufacturing processes [63], this approach imposes a specific limitation for additive manufacturing processes: The thermal complexity of the process along with the complexity of the geometries to be printed renders it impractical to model all known physical phenomena for estimation with state-of-the-art computational capacity [64–66]. This study proposes an application-oriented method that shows residual stress accumulation can be reduced by using a simplified thermal model for preemptive parameter adjustment. Among the existing body of work [34, 62], this study is differentiated with simplified thermal criteria with the simplified-yet-efficient model. Exploring further the limit which is essential for observing and acting upon various process quality objective is an imperative research direction, also for other additive manufacturing modalities.

### Support thickness

The simulation results suggest that increasing the support thickness decreases the maximum thermal difference between the top and bottom of the cantilever. Increasing the standard support thickness of 0.8 mm (used throughout the paper) by 50% reduces the simulated maximum temperature difference by 28%. Although a larger support thickness increases cost due to additional material usage, increased processing time, and increased support removal time, the reduced thermal differences could mitigate part distortion.

### 1D FVM-based thermal model

Scan-resolved part-scale modeling has computation times on the order of hours due to the high spatial and temporal resolution required [48]. This resolution is not necessary for the chosen thermal criterion approach. Thermal circuit networks [49] and implicit finite difference [44] part scale models require significantly less computational time compared to scan-resolved modeling by reducing resolution. The presented finite-volume approach allows for simple implementation of nonlinear physics and does not use superlayers for the control approach.

The chosen approach relies on the specific beam-like geometry, allowing simplification by modeling a representative section and assuming heat flow is primarily vertical, with only lateral conduction into the powder bed. These simplifications are not generally valid for all PBF-LB/M geometries. The proposed model may become inaccurate in geometries such as large cross-sections—where progressive laser exposure creates significant thermal heterogeneity within the solid volume—or in asymmetrical or complex geometries, where variations in lateral heat flow give rise to substantial thermal imbalances. In such cases, a higher fidelity model may be required. Simulating multiple connected slices, as in Ref. [44], could address this limitation with minimal additional effort. Further studies should explore strategic selection criteria for the threshold temperature difference value. In scenarios involving complex-shaped parts, strategies for segmenting the thermal body into multiple distinct regions of interest represent a promising research direction for applying simplified modeling approaches.

Despite its simplistic formulation, the implemented FVM-based model is fast and can effectively capture key thermal dynamics. This efficiency stems from the use of simplified 1D heat flow modeling alongside temporal and spatial adaptivity. While the model achieves high accuracy in the upper layers, where heat flow is predominantly vertical, its accuracy decreases in the lower layers of the beam. The primary source of error is the model’s underestimation of temperature in early layers, which is attributed to the absence of lateral (side-to-side) heat flow modeling. As a result, heat dissipation through the supports is overestimated, leading to faster-than-expected cooling. Additionally, the resistance to heat flow through each slice is underestimated because, in reality, not the entire width of the upper section is effectively used for downward heat transfer. Furthermore, the assumption of purely vertical 1D heat flow holds best at the beam’s center (along the *x*-direction) but does not fully account for lateral heat dissipation near the sides, where conduction towards high-cross-section supports is expected.

The model’s boundary conditions, such as a fixed heat transfer coefficient at the powder boundaries and adiabatic conditions within the support region (without explicit powder temperature modeling), further reduce accuracy. However, given the small powder contact area in the *y*-direction, heat dissipation in this direction is assumed to be minor. Spatial adaptivity introduces small oscillations visible every 10 layers in Fig. 10 (right), but its overall impact is negligible, allowing for improved computational efficiency.

The inverse parameter fitting process introduces the risk of overfitting to the training data (i.e., measured temperatures). A comparison with experimental data from thresholded waiting time tests shows a 36% MAPE for measurements starting at *Z* = 8.28 mm. This discrepancy may indicate either overfitting or altered measurement conditions due to variations in environmental or powder properties between experiments.

Besides the models representitivity of the thermal phenomena during the printing process, multiple points are identified to further improve the model’s implementation. Using a compiled language, optimizing data management, and implementing more efficient integration algorithms could enhance computational performance. The current RK4 method may not be optimal for efficiency [67]; implicit and differentiable algorithms could allow for larger timesteps without uncontrolled error growth [68].

## Conclusion

This study introduced a temperature-difference-based thresholding method to adaptively control dwell time in a feedforward strategy for PBF-LB/M, effectively reducing thermal distortion in a cantilever beam geometry. A simplified, one-dimensional FVM thermal model enabled real-time prediction of vertical temperature profile, guiding IDT adjustments without resorting to complex distortion simulations. Experimental results showed a distortion reduction at the tip of the printed cantilever geometry from 519 to 409 $\upmu$m at the beam tip while maintaining total build time.

The thermal model achieved a 125$\times$ speedup over real-time, capturing dominant vertical heat transfer in a cantilever section by assuming unidirectional flow and employing geometry-adapted boundary conditions. Its simplicity and modularity support future integration of non-linear effects.

Future work should explore the generalizability of the thresholding strategy across diverse process conditions and evaluate broader thermal indicators for distortion control. Extending the model to 3D domains and incorporating explicit distortion mechanics may enhance accuracy but will require managing greater computational complexity.

## Data Availability

No datasets were generated or analysed during the current study.
